# Variation in infection length and superinfection enhance selection efficiency in the human malaria parasite

**DOI:** 10.1038/srep26370

**Published:** 2016-05-19

**Authors:** Hsiao-Han Chang, Lauren M. Childs, Caroline O. Buckee

**Affiliations:** 1 Center for Communicable Disease Dynamics, Department of Epidemiology, Harvard T.H. Chan School of Public Health, Boston, MA 02115, USA

## Abstract

The capacity for adaptation is central to the evolutionary success of the human malaria parasite *Plasmodium falciparum*. Malaria epidemiology is characterized by the circulation of multiple, genetically diverse parasite clones, frequent superinfection, and highly variable infection lengths, a large number of which are chronic and asymptomatic. The impact of these characteristics on the evolution of the parasite is largely unknown, however, hampering our understanding of the impact of interventions and the emergence of drug resistance. In particular, standard population genetic frameworks do not accommodate variation in infection length or superinfection. Here, we develop a population genetic model of malaria including these variations, and show that these aspects of malaria infection dynamics enhance both the probability and speed of fixation for beneficial alleles in complex and non-intuitive ways. We find that populations containing a mixture of short- and long-lived infections promote selection efficiency. Interestingly, this increase in selection efficiency occurs even when only a small fraction of the infections are chronic, suggesting that selection can occur efficiently in areas of low transmission intensity, providing a hypothesis for the repeated emergence of drug resistance in the low transmission setting of Southeast Asia.

Malaria is one of the leading causes of disease and death worldwide with more than 200 million cases each year and about half a million deaths^1^. This high global burden of infection and disease is partly attributable to the unique lifecycle and evolutionary potential of *Plasmodium falciparum* malaria parasites. Although young children and infants in endemic regions often suffer from short-lived infections and disease, bearing the brunt of the mortality, older children and adults develop non-sterilizing immunity to the parasite that results in low density, asymptomatic infections. These infections, which often consist of multiple parasite genotypes, can persist undetected for months and contribute to transmission. As many countries move towards malaria elimination, the role of asymptomatic and chronic infections as reservoirs of transmission and sources of drug resistance becomes an increasingly important consideration.

The dynamics of malaria parasites in the blood are highly variable between individuals, resulting in heterogeneous clinical outcomes and infection lengths^2^,^3^,^4^,^5^. During the acute stage of infection, the parasite population grows exponentially due to repeated rounds of asexual reproduction, sometimes causing severe disease that leads to treatment and clearance or results in death^6^,^7^. During chronic or asymptomatic infections, on the other hand, although the parasite population may initially grow rapidly, it is subsequently controlled by the immune system, with parasite replication persisting for months but with densities that are low, often submicroscopic^8^,^9^,^10^,^11^,^12^. Within a malaria-endemic region, therefore, the infectious reservoir is composed of a mixture of chronically and acutely infected individuals. While little is known about the relationship between transmission intensity and the fraction of asymptomatic infections, recent studies have suggested that – contrary to previous dogma – even in low endemicity settings, low density infections make up a substantial fraction of the infectious reservoir^13^,^14^,^15^.

Another characteristic feature of malaria transmission is the fact that infections in any transmission setting may consist of multiple parasite clones, and infected individuals in high transmission settings likely harbor, and transmit, multiple genotypes. Indeed, it has been suggested that mixed genotype infections may even be more infectious to mosquitoes than single genotype infections^9^,^16^. It has also been hypothesized that multiple parasites may be co-transmitted via mosquitoes^17^,^18^. Indeed, many theoretical frameworks assume that parasite genotypes are sufficiently antigenically divergent that they circulate essentially independently of each other^19^,^20^,^21^,^22^. Frequent superinfection provides opportunities for competition among parasite lineages, whether via cross-reactive immune responses or in competition for red blood cells, both within-host and during transmission. This additional layer of complexity is not easily accommodated in standard theoretical and evolutionary frameworks such as the Wright-Fisher model^23^,^24^.

The impact of these characteristics of malaria infections on the evolutionary capacity of the parasite is unclear, despite the central role that adaptation plays in its epidemiological success. We have previously shown that the within-host parasite population expansion and repeated bottlenecks imposed by the malaria parasite life cycle during acute infections have a profound impact on the population genetics of the parasite^25^,^26^, including reducing the probability of fixation of beneficial mutations and the power to detect positive selection. However, genomic analyses from field isolates were still able to detect genes under positive selection^27^,^28^,^29^,^30^,^31^, and drug resistance has emerged multiple times^32^,^33^,^34^,^35^,^36^. Moreover, the majority of drug resistant parasites have emerged in Southeast Asia^32^,^34^,^35^,^36^, an area of low transmission with an assumed low effective population size, contrary to the predictions of classic population genetic models.

We hypothesize that these paradoxical findings might arise because, in addition to exponential expansion and bottlenecks, chronicity and superinfection create additional differences from the Wright-Fisher model. In particular, chronic infections allow for multiple transmission events over several weeks or months, violating the assumption of “non-overlapping generations” in typical population genetic models^23^,^24^, and different mixtures of short and long infections change the epidemiological dynamics that underlie adaptation. Here, we develop the first stochastic population genetic model that incorporates characteristic features of the malaria parasite lifecycle, including repeated within-host population expansion and between-host bottlenecks, superinfection and variation in infection length, and within-host competition. We show that the probability of fixation of beneficial alleles is higher in chronic infections, and that fixation is more likely-and occurs more rapidly-when superinfection is included. We then analyze a mixed population composed of both acute and chronic infections, which is likely to represent most malaria-endemic settings, and show that having variable infection dynamics increases the probability of fixation of beneficial alleles; unexpectedly, this increase is further enhanced when the proportion of chronic infections is low. Our results have important implications for our understanding the evolutionary dynamics of the malaria parasite, and other pathogens that cause both acute and chronic infections.

## Results

### Chronic infections exhibit higher probability of fixation than acute infections

**Figure 1:**
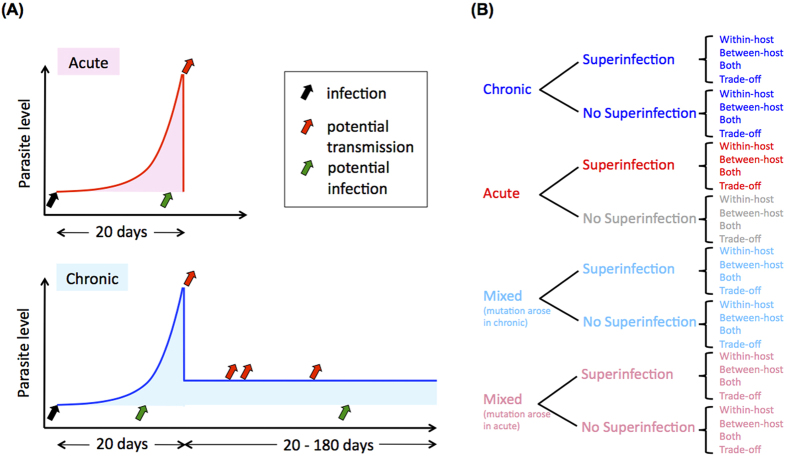
(**A**) The patient models. Acute infections are short and parasites can only be transmitted on day 20 due to the necessary maturation time of parasites; chronic infections are longer and parasites can potentially be transmitted at multiple time points after day 20. (**B**) A list of the models used in this study. Consistent colors (blue for chronic, red for acute) are used throughout the figures.

To characterize the effect of long-lived infections on malaria parasite evolution, we first compare a model with only acute infections to a model with only chronic infections (see Methods). We applied idealized infection models (see Fig. 1A), which reflect trends recorded during experimental infections of humans and observations from field settings^8^,^9^,^37^. In order to compare between models we keep the number of infected human and mosquito hosts constant, which is equivalent to assuming that the prevalence of infection (a common measure of transmission intensity) is at equilibrium. That is, the average number of newly infected hosts in each iteration is equal to the average number of infected hosts who die or clear infections, i.e. an effective reproductive number of unity (*R*_*e*_ = 1). We compare the probability that a beneficial mutation occurring on the same day post-infection in the two models becomes fixed in the population of hosts, as well as the time to reach this fixation. We examine selection of alleles that are beneficial within-host (*s*_*h*_^*+*^*t*_*m*_^*0*^) and during the transmission between hosts (*s*_*h*_^*0*^*t*_*m*_^*+*^) separately, and when they are beneficial both within-host and between-hosts (*s*_*h*_^*+*^*t*_*m*_^*+*^) or are beneficial in one host but deleterious in the other (trade-off models, *s*_*h*_^*−*^*t*_*m*_^*+*^ or *s*_*h*_^*+*^*t*_*m*_^*−*^). Selection coefficients during infection within the host versus between hosts are denoted, respectively, by *s*_*h*_ and *t*_*m*_. We vary *s*_*h*_ between 0.1, 0, and −0.01, corresponding to *s*_*h*_^*+*^, *s*_*h*_^*0*^, and *s*_*h*_^*−*^, and we vary *t*_*m*_ between 0.1, 0, and −0.01, corresponding to *t*_*m*_^*+*^, *t*_*m*_^*0*^, and *t*_*m*_^*−*^, respectively (Table S1).

Chronicity promotes the fixation of beneficial mutations occurring during the exponential growth phase of infection, and the probability of fixation increases with the length of infection, although the time to fixation also increases with infection length (Figs S1A and S2A), consistent with the reduced incidence in populations with longer infections. The selection models incorporating within-host advantage but not between-host disadvantage (*s*_*h*_^*+*^*t*_*m*_^*0*^ and *s*_*h*_^*+*^*t*_*m*_^*+*^) show this pattern, but the model with only between-host advantage (*s*_*h*_^*0*^*t*_*m*_^*+*^ and *s*_*h*_^*−*^*t*_*m*_^*+*^) does not. In selection models incorporating within-host advantage but not between-host disadvantage (*s*_*h*_^*+*^*t*_*m*_^*0*^ and *s*_*h*_^*+*^*t*_*m*_^*+*^), because the frequency of beneficial alleles at the end of infection is greater in the chronic-infection model (Table S2), the probability of passing beneficial alleles to the next host(s) during the transmission bottleneck is greater, and the probability of fixation is greater. In the selection model with only between-host advantage (*s*_*h*_^*0*^*t*_*m*_^*+*^), mutation frequencies within the host neutrally fluctuate during infection, and the probability of fixation is not influenced by the length of infection. In the trade-off model with between-host advantage but within-host disadvantage (*s*_*h*_^*−*^*t*_*m*_^*+*^), the probability of fixation decreases with the length of infection because the frequency of the mutation decreases over time within the host. In the trade-off model with within-host advantage but between-host disadvantage (*s*_*h*_^*+*^*t*_*m*_^*−*^), the probability of fixation is always smaller than 10^−6^ (Fig. S2A). The probability of fixation remains so low because even if a mutation becomes fixed in an individual host, there is selection against transmission of this within-host fixation.

### Superinfection greatly increases selection efficiency

Superinfection dramatically increases the probability of fixation for all selection models except trade-off between-host model (*s*_*h*_^*−*^*t*_*m*_^*+*^) (Fig. S2B). Including superinfection in addition to chronicity in the malaria model leads to a probability of fixation greater than predicted from the Wright-Fisher model^25^, supporting the observations of frequent adaptive evolution in malaria parasites. Here, we assume that superinfection could occur at any point during infection, and originate from any other infectious host. Superinfection does not impact the acute model, although it is included, because parasites from secondary infections are not mature by the time of transmission in acute infections. Superinfection leads to an increased acquisition of infections per host as well as an increased number of outgoing transmissions, and promotes competition between alleles, increasing the probability of fixation in all selection models except the trade-off between-host model (*s*_*h*_^*−*^*t*_*m*_^*+*^). In that model, superinfection increases the competition between alleles within the host, and, because the mutation is deleterious within the host, reduces the probability of fixation.

Interestingly, the speed of fixation shows a complex relationship with infection length and mode of selection. The time to fixation decreases with the length of infection for the models of within-host advantage (*s*_*h*_^*+*^*t*_*m*_^*0*^, *s*_*h*_^*+*^*t*_*m*_^*+*^ and *s*_*h*_^*+*^*t*_*m*_^*−*^) including superinfection (Fig. S2B). In this case, despite the reduced clearance rate, a beneficial allele can be transmitted to both new infections and to existing infections, without “waiting” for clearance of an infection with only the wild-type allele. Because superinfection brings wild-type and mutant alleles together frequently and facilitates direct competition between them, the time to fixation decreases dramatically with superinfection. In the models with between-host advantage but lacking within-host advantage (*s*_*h*_^*0*^*t*_*m*_^*+*^ and *s*_*h*_^*−*^*t*_*m*_^*+*^), the time to fixation increases with the length of infection (Fig. S2B). Since there is no selection advantage or even disadvantage within the host in the models with only between-host advantage (*s*_*h*_^*0*^*t*_*m*_^*+*^ and *s*_*h*_^*−*^*t*_*m*_^*+*^), the frequency of beneficial alleles fluctuates stochastically or decreases within the host. Even if a mutation reaches fixation within the host by chance, superinfection can still subsequently introduce wild-type alleles, lowering the speed of fixation.

### Infection length variation in the host population enhances selection efficiency

The epidemiology of most malaria-endemic regions is characterized by infections of varying lengths. Although the relative proportion of short- and long-lived infections in different transmission settings is poorly understood, it is generally assumed that the fraction of chronic infections will increase with transmission intensity as increasing fractions of the population become semi-immune. We therefore extend our model to investigate the probabilities of fixation in populations with the same number of infected human hosts (that is, the same prevalence) but different proportions of chronic infections. We initially assume equal infectiousness of acute and chronic patients (the default value of the relative infectiousness of acute to chronic infections, *B*, is 1) to reflect the uncertainty about the relationship between parasite density and infectiousness to mosquitoes^13^,^15^,^38^. In the mixed model, we assume that new infections originate from either chronic or acute, and we vary the fraction of chronic infections in the populations.

**Figure 2:**
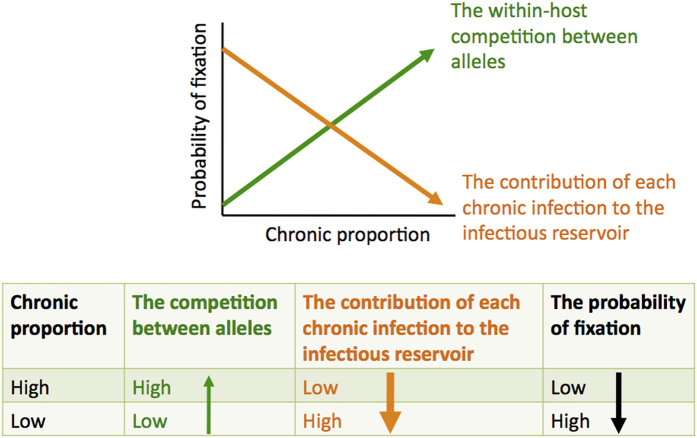
The probability of fixation is affected by both within-host competition and the contribution of each chronic infection to the infectious reservoir in the mixed model. The balance of these two forces depends on selection coefficient and the relative infectiousness of acute and chronic infections.

**Figure 3:**
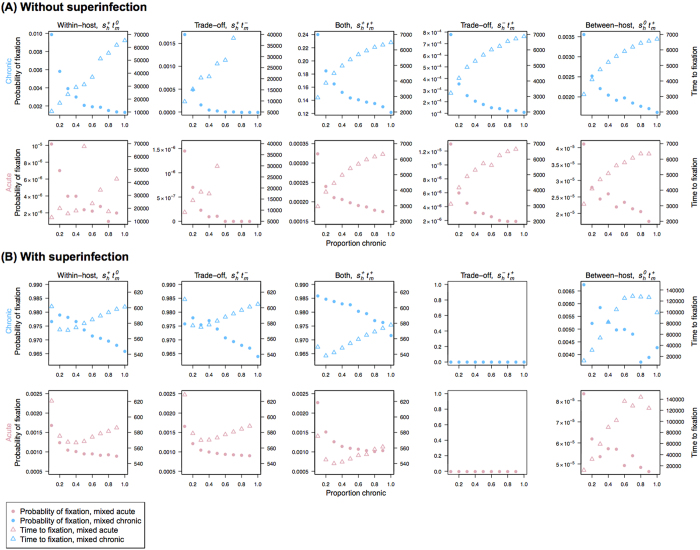
The probability of fixation and the time to fixation in the mixed model, which includes both acute and chronic patients. Without superinfection (**A**) and with superinfection (**B**), the first and second rows show mutations that occurred in chronic (blue) and acute (red) patients, respectively. Triangles represent the time to fixation and circles are the probability of fixation. The type of selection is indicated at the top of each column. The probability of fixation is higher if a beneficial mutation arises in a chronic patient, and tends to decrease with the proportion of chronic patients in the population regardless of whether the mutation happens in an acute or chronic patient. The time to fixation tends to increase with proportion of chronic infections.

In this scenario, two opposing forces shape the probability of fixation (Fig. 2): an increasing fraction of infections that are long-lived enhances allelic competition within the host, but it also changes the dynamics of transmission to reduce the contribution of chronically infected individuals to the infectious reservoir (Fig. S3). As a result, we find that the probability of fixation actually decreases with the fraction of chronic infections, regardless of the inclusion of superinfection in the model (Fig. 3). Here, in the populations where long-lived infections constitute the majority of the infectious reservoir, although within-host competition between alleles is very high, the number of newly infected hosts per unit time is relatively low because most people are already infected (Fig. S3). When the proportion of chronic infections is low, on the other hand, the parasite population benefits both from the increased probability that a beneficial mutation will compete successfully and be selected and transmitted at multiple time points during a long infection, and also from the frequent availability of new susceptible hosts due to cleared acute infections. With superinfection, the contribution of chronically infected individuals to the infectious reservoir decreases less with the proportion of chronic infections, and we observed a smaller change in the probability of fixation with the proportion of chronic infections (Fig. 3B). Assuming that low transmission intensity is associated with a substantial fraction of acute infections due to low population-level immunity^39^, our results imply that we expect the highest probability of fixation of beneficial alleles in regions with low transmission intensity.

This association is not sensitive to the total number of infected human hosts overall or the incidence of infection (Fig. S4), although the model of between-host advantage (*s*_*h*_^*0*^*t*_*m*_^*+*^) is impacted by stochastic fluctuations in allele frequency within the host. We compared two cases with the same level of incidence (i.e., similar numbers of newly infected hosts in each iteration), and the probability of fixation is consistently smaller in the model with larger proportion of chronic infections (Table S3). Given that acute infections may be characterized by high parasite densities and potentially higher infectiousness, we increased the relative infectiousness of acute to chronic infections (*B*). Even when acute infections are twice as infectious as chronic infections, the relationship between the probability of fixation and the proportion of chronic infections remains the same (Fig. S5). Note that if acute infections are orders of magnitude more infectious, we expect the balance of forces shown in Fig. 2 to shift.

Time to fixation showed a relatively complex dependence on the type of selection occurring and the inclusion of superinfection (Fig. 3). Although it was not significantly impacted by whether the beneficial mutation occurred in acute or chronic infections, time to fixation tends to increase with the proportion of chronic infections (Fig. 3). Similar to the results from the chronic-infection only model, superinfection and within-host advantage (*s*_*h*_^*+*^*t*_*m*_^*0*^, *s*_*h*_^*+*^*t*_*m*_^*+*^ and *s*_*h*_^*+*^*t*_*m*_^*−*^) both acted to accelerate the speed of fixation. Within-host advantage increases the frequency of beneficial alleles within the host, and superinfection enhances the number of transmissions per unit time as well as competition between beneficial and wild-type alleles. Again, the mixed model with superinfection but without within-host advantage (*s*_*h*_^*0*^*t*_*m*_^*+*^ and *s*_*h*_^*−*^*t*_*m*_^*+*^) shows the slowest speed of fixation because the mutation fluctuates neutrally or decreases within the host and superinfection acts to slow down the within-host fixation process by bringing wild-type alleles into the host.

## Discussion

**Figure 4:**
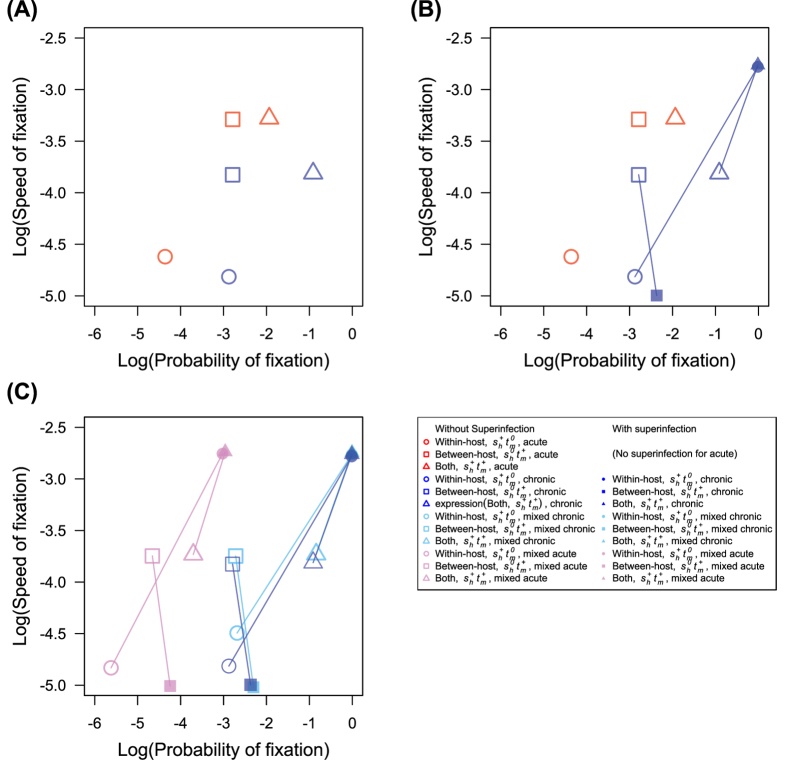
The probability of fixation and the speed of fixation. The speed of fixation is equal to the inverse of the time to fixation. This plot is shown in log-log scale. (**A**) Chronic infections (blue symbols) have higher probability of fixation (except for the between host *s*_*h*_^*0*^*t*_*m*_^*+*^ model) while acute infections (red symbols) have higher speed of fixation. (**B**) Superinfection (filled symbols) greatly increases both the probability and the speed of fixation in chronic models containing within-host advantage but not between-host disadvantage (*s*_*h*_^*+*^*t*_*m*_^*0*^ and *s*_*h*_^*+*^*t*_*m*_^*+*^), while decreasing speed of fixation and increasing probability of fixation in between-host model (*s*_*h*_^*0*^*t*_*m*_^*+*^). Identical model types with and without superinfection are joined by a line. (**C**) The mixed model, with both acute and chronic infections in the same population, increases both speed of and probability of fixation of mutations occurring in chronic infections (the values shown here are from the mixed model with 50% chronic infections). The effect of superinfection in the mixed model is similar to that in the chronic-infection only model. Pink and cyan indicate the mixed model with a beneficial mutation occurring in acute and chronic patients, respectively.

We have shown using a population genetic model that several aspects of the lifecycle of *Plasmodium falciparum*, namely chronicity and superinfection, combine to enhance selection efficiency for beneficial mutations, particularly for mutations conferring advantage within the host. Mutations occurring in the chronic-infection only model offering within-host advantage have higher probability of fixation than those occurring in the acute-infection only model (Fig. 4A). Superinfection further increases selection efficiency of mutations occurring in chronic patients, making both probability and speed of fixation higher in the chronic-infection model (Fig. 4B). Superinfection also dramatically increases the probability of fixation in the model with acute and chronic infections (Fig. 4C). Interestingly, selection is most efficient in the model with both acute and chronic infections, and the association between the probability of fixation and the proportion of chronic infections is negative (Fig. 3).

Our results imply that ignoring superinfection and overlapping generations due to the variation in duration of infection strongly biases our understanding and prediction of adaptation of malaria parasites. For instance, previous models ignoring superinfection and chronicity predicted a lower probability of fixation for beneficial mutations and lower ability to detect positive selection compared to the Wright-Fisher model^25^,^26^, while empirical genomic analysis detected signals of positive selection. Including both chronicity and superinfection in our model leads to a much greater probability of fixation, reconciling the discrepancy between model predictions and observations.

These results have important implications for the emergence of drug resistance mutations. Resistance to chloroquine, sulphadoxine-pyrimethamine, mefloquine, and artemisinin all emerged in Southeast Asia, a relatively low transmission setting, where we would expect a lower parasite effective population size than in Africa^32^,^34^,^35^,^36^,^40^. It has been suggested that the prevalence of counterfeit drugs and frequent use of anti-malarials in Southeast Asia, as well as their earlier introduction, may have led to repeated drug resistance emergence in this region^33^,^41^,^42^. There have been several theoretical studies on the evolution of drug resistance, focusing on different aspects of complexities of malaria infections, including chronicity and superinfection^43^,^44^,^45^,^46^,^47^,^48^,^49^,^50^.

Our study is the first stochastic population genetic model that considers the details of the malaria lifecycle (repeated within-host population expansion and between-host transmission bottlenecks), variation in infection length, superinfection, and within-host competition at the same time. Both the probability and speed and fixation per resistant mutation and the number of new or existing drug resistant mutations are key components of the evolution of drug resistance. We focus on the probability and speed of fixation of a beneficial mutation that may or may not have a trade-off, and our results suggest that these may actually be higher in low transmission settings, if we assume the positive correlation between the proportion of chronic infections and transmission intensity. Here, beneficial mutations have the advantage of rapid spread due to the rapid turnover of acute infections (Fig. S3), and within-host selection of chronic patients if the mutation is beneficial within the host.

It is expected that parasites causing acute infections are likely to be under the strongest selection, since they may produce severe disease requiring treatment, but even for beneficial mutations occurring in acute patients, the combination of chronic infections and superinfection dramatically increases the speed of fixation of mutations offering within-host advantage. Furthermore, the proportion of infections that are treated in low transmission settings is also likely to be higher than that in high transmission settings^51^ because low exposure of malaria infections is thought to lead to low immunity and higher proportion of clinical symptoms, further increasing selection pressure on the parasite population. Our results therefore provide a mechanistic hypothesis for why the emergence drug resistance has occurred repeatedly in low transmission settings.

## Methods

We use a stochastic population genetic framework to model within-host allele frequencies in human and mosquito hosts including transmission events between hosts (Fig. S6). Each host is comprised of a population of parasites, and selection can happen within the host, during the transmission or both. Only infected hosts and mosquitoes are included in the model and resolution of one infection on average results in the appearance of a new infection. The parameters and their baseline values are summarized in Table S1.

### Patient model

We assume that during an acute infection, the parasite population expands exponentially to the order of 10^11^ (details below) and either the patient dies or the parasite population is cleared on day 20 (Fig. 1). For chronic infections, the parasite population initially expands as in acute infections, but then declines precipitously to 10^6^ on day 20 and is controlled by the immune system, persisting at this low level for another 20 to 180 days^3^,^52^,^53^. In the mixed model with both acute and chronic infections, for simplicity, we set the length of chronic infection to 200 days.

### Simulation

Consistent with the erythrocytic cycle of *Plasmodium falciparum* in the blood, we used 48 hours as the time unit for one iteration. We assume the number of infected human hosts is *N* = 1000. To reflect an endemic population where individuals are at different stages of infection, we start the simulation assuming an equal number of individuals on each day of infection (as shown in Fig. S3). Initially, all parasites have wild-type alleles. During each iteration, the parasite population size in each host increases, decreases, or stays the same, depending on the day since infection. Similar to previous work^26^, five selection models are used: within-host advantage (*s*_*h*_^*+*^*t*_*m*_^*0*^), between-host advantage (*s*_*h*_^*0*^*t*_*m*_^*+*^), both within- and between-host advantage (*s*_*h*_^*+*^*t*_*m*_^*+*^), and trade-off (*s*_*h*_^*−*^*t*_*m*_^*+*^ or *s*_*h*_^*+*^*t*_*m*_^*−*^). Selection coefficients within the host and during transmission are denoted by *s*_*h*_ and *t*_*m*_, respectively. Results were obtained from at least 10^6^ repeat stochastic simulations or 10,000 fixations, whichever was reached first.

#### Human infections

During infection between days 0 to 18, the parasite population size increases by an average of 16*P*(1 + *s*_*h*_) fold every other day, i.e. each parasite reproduces *X* parasites where *X* is Poisson distributed with mean 16*P*(1 + *s*_*h*_), *P* = 0.9 is the probability of death for each parasite^54^,^55^, and *s*_*h*_ is the selection coefficient within the human host. On infection day 20, the parasite population size is on the order of 10^11^ (assuming *s*_*h*_ = 0, 10*(16*0.9)^9^ = 2.66 × 10^11^)^56^ and is composed of mature parasites that can be transmitted to mosquitoes. During transmission events, a bottleneck occurs in which only *D* = 10 parasites are transmitted to the mosquito^56^.

In order to keep the host population size stable, the number of transmissions is Poisson distributed with the mean equal to *dA*/*r*_*t*_, where *d* represents the expected number of human hosts whose parasites are cleared (*d* = *N*(1−*X*)/*G*_*1*_ + *N*X/*G*_*2*_, where *X* is the proportion of chronic infections, *G*_*1*_ and *G*_*2*_ are duration of infection for acute and chronic infections, respectively), *r*_*t*_ is the number of hosts that can transmit parasites, and *A* = 10 is the ratio of mosquito to human hosts^25^. This implies that the mean number of infections in previously uninfected hosts generated by each infectious host per unit of time decreases with the number of hosts that can transmit parasites. If there is transmission selection (*t*_*m*_ ≠ 0), the number of transmissions is Poisson distributed with the mean (1 + *t*_*m*_)*dAC_*1*_/*r*_*t*_ or *dAC*_*1*_/*r*_*t*_, depending on whether the hosts contain parasites with the mutation or not. *C*_*1*_ is the normalization constant that is used for keeping stable host population size (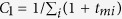, where *t*_*mi*_ represents selection coefficient during transmission from human *i* to mosquito). On day 20, the parasite is cleared in acute infections and the parasite population size decreases to 10^6^ in chronic infections^57^.

In chronic infections, during day 22 and onwards, the parasite population size stays at 10^6^ in chronic infections and evolves by sampling with replacement from the parasites in the previous iteration like the in Wright-Fisher model^23^,^24^. Parasites with or without the mutation have the probability of (1 + *s*_*h*_)*C_*2*_ or *C*_*2*_ to reproduce, where *C*_*2*_ is the normalizing constant that is used to keep stable parasite population size in the human hosts when there is host selection (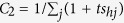, where *s*_*hj*_ represents selection coefficient of parasite *j* within the human host). Parasites can be transmitted to mosquitoes during chronic infections at any iteration starting on day 20.

#### Mosquito infections

In the mosquito, during infection between days 0 and 10, the parasite population replicates 12 times by on average 2*P fold to reach approximately 10*(2*0.9)^12^ = 11568 parasites at the end of the time in the mosquito^55^,^56^,^58^. The incubation period in the mosquito is assumed to be 10 days^59^,^60^, at which point parasites can be transmitted to human host. The bottleneck size during this transmission event is *D* = 10^55^,^56^,^61^. Here, the probability of transmission is the ratio of human to mosquito hosts (1/*A*). Because malaria parasites spend more time in the life cycle in human host, we choose to focus on mutations that are advantageous within the human host or during the transmission from human to mosquito host in this study and assume that the mutation does not have an effect in the mosquito, or during the transmission between the mosquito and human host.

### Mutation and selection

Our model is a one-locus model. The same as the commonly used infinite-site model in population genetics^62^, we assume that mutation for this locus only occurs once. Mutation can take place in any iteration within patients. When comparing acute and chronic models, mutations occurring on the same day post-infection are used. In the within-host selection model, mutant parasites have a (1 + *s*_*h*_) fold higher probability of reproducing within human hosts in each asexual generation than wild-type parasites. In the between-host selection model, hosts with mutant parasites have a (1 + *t*_*m*_) fold higher probability of transmission than hosts with only wild-type parasites. In the model with both within-host and between-host selection, the same mutation has effects on both the probability of reproducing within human hosts and the probability of transmission. We choose to compare the probability of fixation and the time to fixation of a beneficial mutation occurring at day 0 in the human host between models because parasite numbers are smaller in initial days post-infection and mutations have larger frequency in the hosts so that the probability of fixation is higher (Table S2) and there needs smaller number of simulation runs to obtain the estimate of probability of fixation.

### Superinfection

We assume the number of infections is proportional to the duration of infection. Superinfection can happen at any iteration, but can only contribute to outgoing transmissions if they become mature (reach day 20) by the time of transmission. Therefore, superinfection does not have an effect in the acute model as only the initial infection has time to mature. In the patient with superinfection, when the later infection reaches day 20, allele frequencies of the existing infection and the later infection are mixed in the way that the contribution of the later infection to within-host allele frequency is one-tenth of the contribution of the existing infection. For the case with more than two infections, the existing infection is composed of all previous infections.

## Additional Information

**How to cite this article**: Chang, H.-H. *et al*. Variation in infection length and superinfection enhance selection efficiency in the human malaria parasite. *Sci. Rep.* **6**, 26370; doi: 10.1038/srep26370 (2016).

## Supplementary Material

Supplementary Information

## References

[b1] World Health Organization. World Malaria Report 2015. (World Health Organization, Geneva, Switzerland, 2015).

[b2] Bottius E. . Malaria: even more chronic in nature than previously thought; evidence for subpatent parasitaemia detectable by the polymerase chain reaction. Trans. R. Soc. Trop. Med. Hyg. 90, 15–19 (1996). 8730301 10.1016/s0035-9203(96)90463-0

[b3] Bretscher M. T. . The distribution of Plasmodium falciparum infection durations. Epidemics 3, 109–118, doi: 10.1016/j.epidem.2011.03.002 (2011). 21624782

[b4] Childs L. M. & Buckee C. O. Dissecting the determinants of malaria chronicity: why within-host models struggle to reproduce infection dynamics. J. R. Soc. Interface. 12, 20141379, doi: 10.1098/rsif.2014.1379 (2015). 25673299 PMC4345506

[b5] Laishram D. D. . The complexities of malaria disease manifestations with a focus on asymptomatic malaria. Malar. J. 11, 29, doi: 10.1186/1475-2875-11-29 (2012). 22289302 PMC3342920

[b6] Mancio-Silva L., Lopez-Rubio J. J., Claes A. & Scherf A. Sir2a regulates rDNA transcription and multiplication rate in the human malaria parasite Plasmodium falciparum. Nat. Commun. 4, 1530, doi: 10.1038/ncomms2539 (2013). 23443558 PMC3586713

[b7] Simpson J. A., Aarons L., Collins W. E., Jeffery G. M. & White N. J. Population dynamics of untreated Plasmodium falciparum malaria within the adult human host during the expansion phase of the infection. Parasitology 124, 247–263 (2002). 11922427 10.1017/s0031182001001202

[b8] Felger I. . The dynamics of natural Plasmodium falciparum infections. PloS one 7, e45542, doi: 10.1371/journal.pone.0045542 (2012). 23029082 PMC3445515

[b9] Nassir E. . Impact of genetic complexity on longevity and gametocytogenesis of Plasmodium falciparum during the dry and transmission-free season of eastern Sudan. Int. J. Parasitol. 35, 49–55, doi: 10.1016/j.ijpara.2004.10.014 (2005). 15619515

[b10] Langhorne J., Ndungu F. M., Sponaas A. M. & Marsh K. Immunity to malaria: more questions than answers. Nat. Immunol. 9, 725–732, doi: 10.1038/ni.f.205 (2008). 18563083

[b11] Mosha J. F. . Epidemiology of subpatent Plasmodium falciparum infection: implications for detection of hotspots with imperfect diagnostics. Malar. J. 12, 221, doi: 10.1186/1475-2875-12-221 (2013). 23815811 PMC3701503

[b12] Thomas C. J. & Lindsay S. W. Local-scale variation in malaria infection amongst rural Gambian children estimated by satellite remote sensing. Trans. R. Soc. Trop. Med. Hyg. 94, 159–163 (2000). 10897355 10.1016/s0035-9203(00)90257-8

[b13] Bousema T., Okell L., Felger I. & Drakeley C. Asymptomatic malaria infections: detectability, transmissibility and public health relevance. Nat. Rev. Microbiol. 12, 833–840, doi: 10.1038/nrmicro3364 (2014). 25329408

[b14] Okell L. C. . Factors determining the occurrence of submicroscopic malaria infections and their relevance for control. Nat. Commun. 3, 1237, doi: 10.1038/ncomms2241 (2012). 23212366 PMC3535331

[b15] Lindblade K. A., Steinhardt L., Samuels A., Kachur S. P. & Slutsker L. The silent threat: asymptomatic parasitemia and malaria transmission. Expert Rev. Anti. Infect. Ther. 11, 623–639, doi: 10.1586/eri.13.45 (2013). 23750733

[b16] Taylor L. H., Walliker D. & Read A. F. Mixed-genotype infections of the rodent malaria Plasmodium chabaudi are more infectious to mosquitoes than single-genotype infections. Parasitology 115 (Pt 2), 121–132 (1997). 10190168 10.1017/s0031182097001145

[b17] Nkhoma S. C. . Close kinship within multiple-genotype malaria parasite infections. Proc. Biol. Sci. 279, 2589–2598, doi: 10.1098/rspb.2012.0113 (2012). 22398165 PMC3350702

[b18] Sutton P. L., Torres L. P. & Branch O. H. Sexual recombination is a signature of a persisting malaria epidemic in Peru. Malar. J. 10, 329, doi: 10.1186/1475-2875-10-329 (2011). 22039962 PMC3231964

[b19] Buckee C., Danon L. & Gupta S. Host community structure and the maintenance of pathogen diversity. Proc. Biol. Sci. 274, 1715–1721, doi: 10.1098/rspb.2007.0415 (2007). 17504739 PMC2493584

[b20] Buckee C. O., Koelle K., Mustard M. J. & Gupta S. The effects of host contact network structure on pathogen diversity and strain structure. Proc. Natl. Acad. Sci. USA 101, 10839–10844, doi: 10.1073/pnas.0402000101 (2004). 15247422 PMC490021

[b21] Griffin J. T. . Reducing Plasmodium falciparum malaria transmission in Africa: a model-based evaluation of intervention strategies. PLoS Med. 7, doi: 10.1371/journal.pmed.1000324 (2010). PMC2919425 20711482

[b22] Gupta S., Ferguson N. & Anderson R. Chaos, persistence, and evolution of strain structure in antigenically diverse infectious agents. Science 280, 912–915 (1998). 9572737 10.1126/science.280.5365.912

[b23] Fisher R. A. The genetical theory of natural selection. (Clarendon Press, 1930).

[b24] Wright S. Evolution in Mendelian Populations. Genetics 16, 97–159 (1931). 17246615 10.1093/genetics/16.2.97 PMC1201091

[b25] Chang H. H. . Malaria life cycle intensifies both natural selection and random genetic drift. Proc. Natl. Acad. Sci. USA 110, 20129–20134, doi: 10.1073/pnas.1319857110 (2013). 24259712 PMC3864301

[b26] Chang H. H. & Hartl D. L. Recurrent bottlenecks in the malaria life cycle obscure signals of positive selection. Parasitology 142 Suppl 1, S98–S107, doi: 10.1017/S0031182014000067 (2015). 24560397 PMC4139472

[b27] Amambua-Ngwa A. . SNP genotyping identifies new signatures of selection in a deep sample of West African Plasmodium falciparum malaria parasites. Mol. Biol. Evol. 29, 3249–3253, doi: 10.1093/molbev/mss151 (2012). 22688945 PMC3472499

[b28] Amambua-Ngwa A. . Population genomic scan for candidate signatures of balancing selection to guide antigen characterization in malaria parasites. PLoS Genet. 8, e1002992, doi: 10.1371/journal.pgen.1002992 (2012). 23133397 PMC3486833

[b29] Chang H. H. . Genomic sequencing of Plasmodium falciparum malaria parasites from Senegal reveals the demographic history of the population. Mol. Biol. Evol. 29, 3427–3439, doi: 10.1093/molbev/mss161 (2012). 22734050 PMC3472501

[b30] Mobegi V. A. . Genome-wide analysis of selection on the malaria parasite Plasmodium falciparum in West African populations of differing infection endemicity. Mol. Biol. Evol. 31, 1490–1499, doi: 10.1093/molbev/msu106 (2014). 24644299 PMC4032133

[b31] Park D. J. . Sequence-based association and selection scans identify drug resistance loci in the Plasmodium falciparum malaria parasite. Proc. Natl. Acad. Sci. USA 109, 13052–13057, doi: 10.1073/pnas.1210585109 (2012). 22826220 PMC3420184

[b32] Hurwitz E. S., Johnson D. & Campbell C. C. Resistance of Plasmodium falciparum malaria to sulfadoxine-pyrimethamine (‘Fansidar’) in a refugee camp in Thailand. Lancet 1, 1068–1070 (1981). 6112445 10.1016/s0140-6736(81)92239-x

[b33] Mita T. & Tanabe K. Evolution of Plasmodium falciparum drug resistance: implications for the development and containment of artemisinin resistance. Jpn. J. Infect. Dis. 65, 465–475 (2012). 23183197 10.7883/yoken.65.465

[b34] Wernsdorfer W. H. & Payne D. The dynamics of drug resistance in Plasmodium falciparum. Pharmacol. Ther. 50, 95–121 (1991). 1891480 10.1016/0163-7258(91)90074-v

[b35] Wongsrichanalai C., Pickard A. L., Wernsdorfer W. H. & Meshnick S. R. Epidemiology of drug-resistant malaria. Lancet Infect. Dis. 2, 209–218 (2002). 11937421 10.1016/s1473-3099(02)00239-6

[b36] Young M. D. & Moore D. V. Chloroquine resistance in Plasmodium falciparum. Am. J. Trop. Med. Hyg. 10, 317–320 (1961). 13787478 10.4269/ajtmh.1961.10.317

[b37] Collins W. E. & Jeffery G. M. A retrospective examination of secondary sporozoite- and trophozoite-induced infections with Plasmodium falciparum: development of parasitologic and clinical immunity following secondary infection. Am. J. Trop. Med. Hyg. 61, 20–35 (1999). 10432042 10.4269/tropmed.1999.61-020

[b38] Churcher T. S. . Predicting mosquito infection from Plasmodium falciparum gametocyte density and estimating the reservoir of infection. eLife 2, e00626, doi: 10.7554/eLife.00626 (2013). 23705071 PMC3660740

[b39] Doolan D. L., Dobano C. & Baird J. K. Acquired immunity to malaria. Clin. Microbiol. Rev. 22, 13–36, Table of Contents, doi: 10.1128/CMR.00025-08 (2009). 19136431 PMC2620631

[b40] Noedl H. . Evidence of artemisinin-resistant malaria in western Cambodia. N. Engl. J. Med. 359, 2619–2620, doi: 10.1056/NEJMc0805011 (2008). 19064625

[b41] Maude R. J., Woodrow C. J. & White L. J. Artemisinin Antimalarials: Preserving the “Magic Bullet”. Drug Dev. Res. 71, 12–19, doi: 10.1002/ddr.20344 (2010). 21399699 PMC3048293

[b42] Rathod P. K., McErlean T. & Lee P. C. Variations in frequencies of drug resistance in Plasmodium falciparum. Proc. Natl. Acad. Sci. USA 94, 9389–9393 (1997). 9256492 10.1073/pnas.94.17.9389 PMC23200

[b43] Antao T. & Hastings I. M. Environmental, pharmacological and genetic influences on the spread of drug-resistant malaria. Proc. Biol. Sci. 278, 1705–1712, doi: 10.1098/rspb.2010.1907 (2011). 21084349 PMC3081768

[b44] Hastings I. M. Complex dynamics and stability of resistance to antimalarial drugs. Parasitology 132, 615–624, doi: 10.1017/S0031182005009790 (2006). 16426485

[b45] Hastings I. M. & Watkins W. M. Intensity of malaria transmission and the evolution of drug resistance. Acta Trop. 94, 218–229, doi: 10.1016/j.actatropica.2005.04.003 (2005). 15847846

[b46] Kim Y., Escalante A. A. & Schneider K. A. A population genetic model for the initial spread of partially resistant malaria parasites under anti-malarial combination therapy and weak intrahost competition. PloS one 9, e101601, doi: 10.1371/journal.pone.0101601 (2014). 25007207 PMC4090191

[b47] Klein E. Y., Smith D. L., Boni M. F. & Laxminarayan R. Clinically immune hosts as a refuge for drug-sensitive malaria parasites. Malar. J. 7, 67, doi: 10.1186/1475-2875-7-67 (2008). 18439283 PMC2409364

[b48] Klein E. Y., Smith D. L., Laxminarayan R. & Levin S. Superinfection and the evolution of resistance to antimalarial drugs. Proc. Biol. Sci. 279, 3834–3842, doi: 10.1098/rspb.2012.1064 (2012). 22787024 PMC3415915

[b49] Mackinnon M. J. Drug resistance models for malaria. Acta Trop. 94, 207–217, doi: 10.1016/j.actatropica.2005.04.006 (2005). 15894181

[b50] Pongtavornpinyo W. . Probability of emergence of antimalarial resistance in different stages of the parasite life cycle. Evol. Appl. 2, 52–61, doi: 10.1111/j.1752-4571.2008.00067.x (2009). 20526409 PMC2880443

[b51] Talisuna A. O., Okello P. E., Erhart A., Coosemans M. & D’Alessandro U. Intensity of malaria transmission and the spread of Plasmodium falciparum resistant malaria: a review of epidemiologic field evidence. Am. J. Trop. Med. Hyg. 77, 170–180 (2007). 18165490

[b52] Eckhoff P. P. falciparum infection durations and infectiousness are shaped by antigenic variation and innate and adaptive host immunity in a mathematical model. PloS one 7, e44950, doi: 10.1371/journal.pone.0044950 (2012). 23028698 PMC3446976

[b53] Sama W., Owusu-Agyei S., Felger I., Vounatsou P. & Smith T. An immigration-death model to estimate the duration of malaria infection when detectability of the parasite is imperfect. Stat. Med. 24, 3269–3288, doi: 10.1002/sim.2189 (2005). 16143990

[b54] Cheng Q. . Measurement of Plasmodium falciparum growth rates *in vivo*: a test of malaria vaccines. Am. J. Trop. Med. Hyg. 57, 495–500 (1997). 9347970 10.4269/ajtmh.1997.57.495

[b55] White N. J. . Malaria. Lancet 383, 723–735, doi: 10.1016/S0140-6736(13)60024-0 (2014). 23953767

[b56] Kappe S. H., Vaughan A. M., Boddey J. A. & Cowman A. F. That was then but this is now: malaria research in the time of an eradication agenda. Science 328, 862–866, doi: 10.1126/science.1184785 (2010). 20466924

[b57] Dietz K., Raddatz G. & Molineaux L. Mathematical model of the first wave of Plasmodium falciparum asexual parasitemia in non-immune and vaccinated individuals. Am. J. Trop. Med. Hyg. 75, 46–55 (2006). 16931815 10.4269/ajtmh.2006.75.46

[b58] Rosenberg R. & Rungsiwongse J. The number of sporozoites produced by individual malaria oocysts. Am. J. Trop. Med. Hyg. 45, 574–577 (1991). 1951866 10.4269/ajtmh.1991.45.574

[b59] Baton L. A. & Ranford-Cartwright L. C. Spreading the seeds of million-murdering death: metamorphoses of malaria in the mosquito. Trends Parasitol. 21, 573–580, doi: 10.1016/j.pt.2005.09.012 (2005). 16236552

[b60] Rosenberg R., Wirtz R. A., Schneider I. & Burge R. An estimation of the number of malaria sporozoites ejected by a feeding mosquito. Trans. R. Soc. Trop. Med. Hyg. 84, 209–212 (1990). 2202101 10.1016/0035-9203(90)90258-g

[b61] Medica D. L. & Sinnis P. Quantitative dynamics of Plasmodium yoelii sporozoite transmission by infected anopheline mosquitoes. Infect. Immun. 73, 4363–4369, doi: 10.1128/IAI.73.7.4363-4369.2005 (2005). 15972531 PMC1168603

[b62] Kimura M. The number of heterozygous nucleotide sites maintained in a finite population due to steady flux of mutations. Genetics 61, 893–903 (1969). 5364968 10.1093/genetics/61.4.893 PMC1212250

