# Supplementary material for: Variation in infection length and superinfection enhance selection efficiency in the human malaria parasite
Source: Sci Rep. 2016 May 19;6:26370. doi: 10.1038/srep26370 (PMC4872237; doi:10.1038/srep26370)
Supplement: Supplementary Information [file srep26370-s1.pdf]

Title: Variation in infection length and superinfection enhance selection efficiency in the human malaria parasite

Hsiao-Han Chang, Lauren M. Childs, and Caroline O. Buckee

Center for Communicable Disease Dynamics, Department of Epidemiology, Harvard T.H. Chan School of Public Health, Boston, MA 02115, USA

Supplementary materials

Supplementary text

Model assumptions and implications

The number of infected hosts does not vary with the proportion of chronic infections

It has become increasingly clear that the asymptomatic reservoir for malaria transmission is substantial, even in relatively low transmission settings[1-4]. Although it is generally assumed that fewer infections are asymptomatic at low transmission intensity, due to limited herd immunity, the impact of transmission setting on the evolution of the parasite is key to understanding its response to interventions. Therefore, we hold the number of infected hosts constant, and vary the proportion that are chronic, to explore the impact of long-lived infections on parasite evolution. We assume that the number of infected hosts is at equilibrium and does not vary with the proportion of chronic infections in order to make a comparison of the probability of fixation across models. It is known that the probability of fixation is sensitive to the initial allele frequency in the population due to the effect of genetic drift, and the initial allele frequency is determined by the total number of infected hosts in the model (Figure S4). It is expected that the probability of fixation is smaller when the number of infected hosts is larger in the case of neutral mutation. However, in the case when mutation is not neutral, as we are considering, there is no simple association with the size of the population. Because our goal is to study the effect of variation in infection length on selection but not the effect of initial allele frequency, we control for the number of infected hosts across models.

Simplifying within-host dynamics

Simplifying within-host dynamics is standard in the population genetic models of malaria parasites to make simulations computationally tractable [5-8]. In our previous work, we showed that genetic drift and selection are both affected by repeated within-host expansion and between-host bottlenecks [9,10]. To study the effect of variation in infection length on the efficiency of selection, we include the repeated within-host expansion and between-host bottlenecks.

We assume that parasites undergo 12 replication cycles in the mosquito to reach the parasite population size of ~10^4 after 10 days. Because expansion of the parasite in the oocyst is not well understood and the number of sporozoites (in the order of 10^4) and the number of days it takes to produce sporozoites period (10 days) are known (Table S1), we assume 12 replications in 10 days to fit the number of sporozoites that is known. Because we assume the mutation is neutral within the mosquito host, the number of replications in the mosquito host is not expected to change the results qualitatively.

We do not differentiate liver and blood stages of malaria parasites, and simplify the replication process within the human host by assuming that each replication leads to in average 16*0.9 parasites. If we were to include different parasite stages in the model, we expect the magnitude of our results to be shifted slightly, but the relative relationship between models will not be affected.

Same infectiousness of chronic and acute patients

We assume chronic and acute patients have the same infectiousness because the relationship between parasite density and infectiousness to mosquitoes is uncertain [1,2,11]. We showed that even when acute infections are twice as infectious as chronic infections, the

relationship between the probability of fixation and the proportion of chronic infections remains the same (Fig. S5). If acute infections are orders of magnitude more infectious, we expect the balance of forces shown in Fig. 2 to shift.

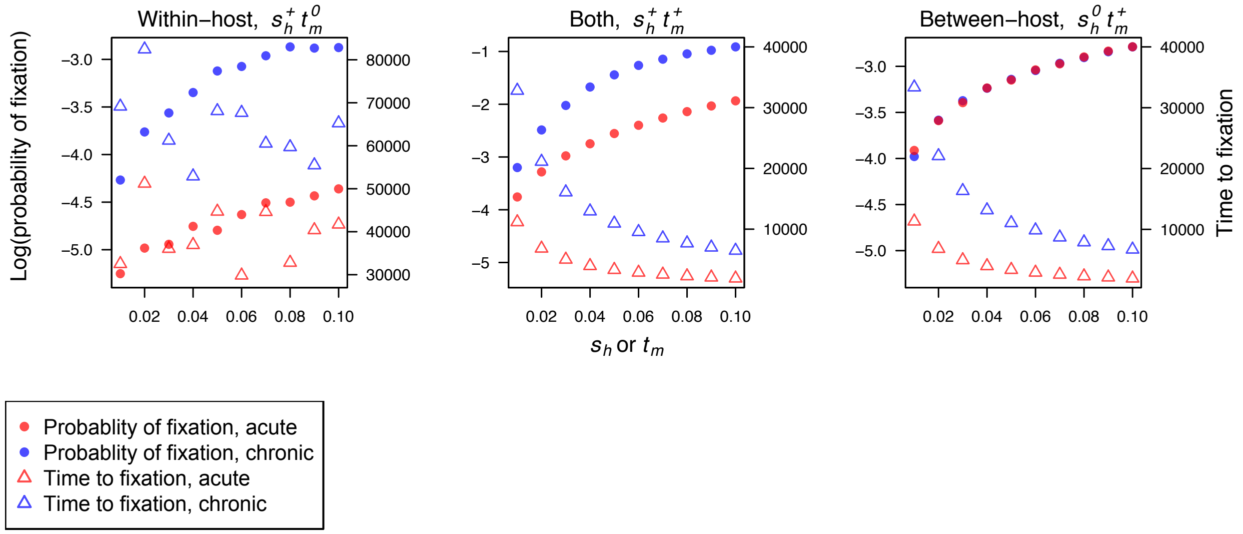

Supplementary Figure S1. The probability of fixation and the time to fixation in acute-infection and chronic-infection models as selective force varies. The probability of fixation is higher in the chronic model (blue symbols) than in the acute model (red symbols), except for between-host ($s_h^0 t_m^+$) model, and increases with as the selection coefficient increases. The time to fixation is higher in the chronic model than in the acute model and decreases with the selection coefficient except for when there is only within-host advantage ($s_h^+ t_m^0$). In the chronic model, the length of infection is assumed to be 200 days.

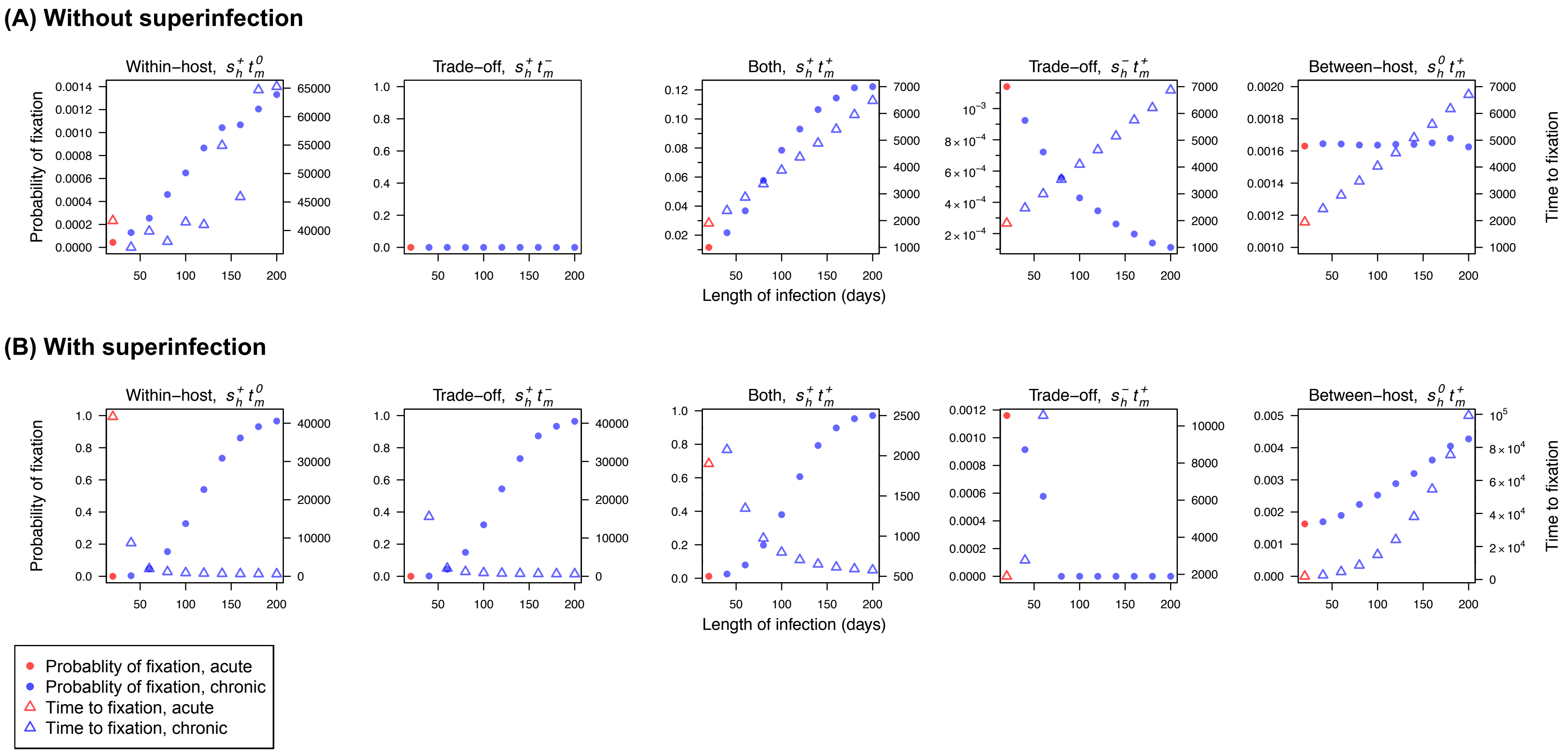

Supplementary Figure S2. The probability of fixation and the time to fixation in the short acute-infection and varying length chronic-infection models. (A) The probability of fixation (solid circles) increases with the duration of the chronic infection when the mutation is beneficial within the host ($s_h^+t_m^0$ and $s_h^+t_m^+$), but does not change or decreases when the mutation is beneficial during the transmission ($s_h^0t_m^+$ and $s_h^-t_m^+$). The time to fixation (triangles) increases with duration of infection in all models. **(B)** Incorporating superinfection greatly enhances (note different y axes) the probability of fixation in the chronic-infection model (blue symbols) compared to (A). With superinfection, the time to fixation decreases with the duration of infection in cases where the mutation is beneficial within the host ($s_h^+t_m^0$, $s_h^+t_m^+$, and $s_h^+t_m^-$); the time to fixation increases with the length of infection when the mutation is only advantageous during transmission but not within the host ($s_h^0t_m^+$ and $s_h^-t_m^+$).

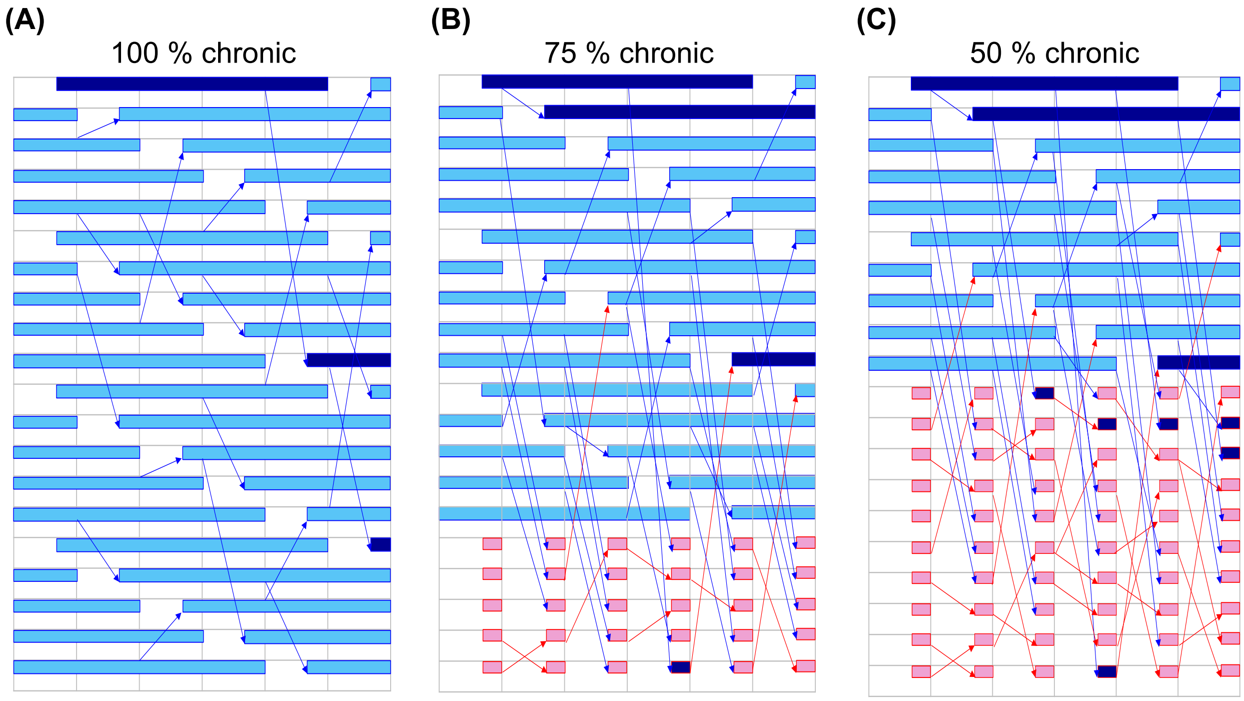

Supplementary Figure S3. Illustration of transmission dynamics in models with different proportion of chronic infections. The proportion of chronic infections **(A)** 100 % **(B)** 75 % and **(C)** 50 % where light blue and red boxes indicate chronic and acute infections, respectively. Blue and red lines represent infections transmitted from chronic and acute infections, respectively. Dark blue box indicates hosts with mutations that originally arose in chronic infections. The population with a lower proportion of chronic infections has a higher turnover rate. Thus, a mutation occurring in chronic infections has a higher chance to be transmitted and increase in frequency through rapidly cleared acute infections. This figure is a simplified example to help build intuition on the dynamics of the system.

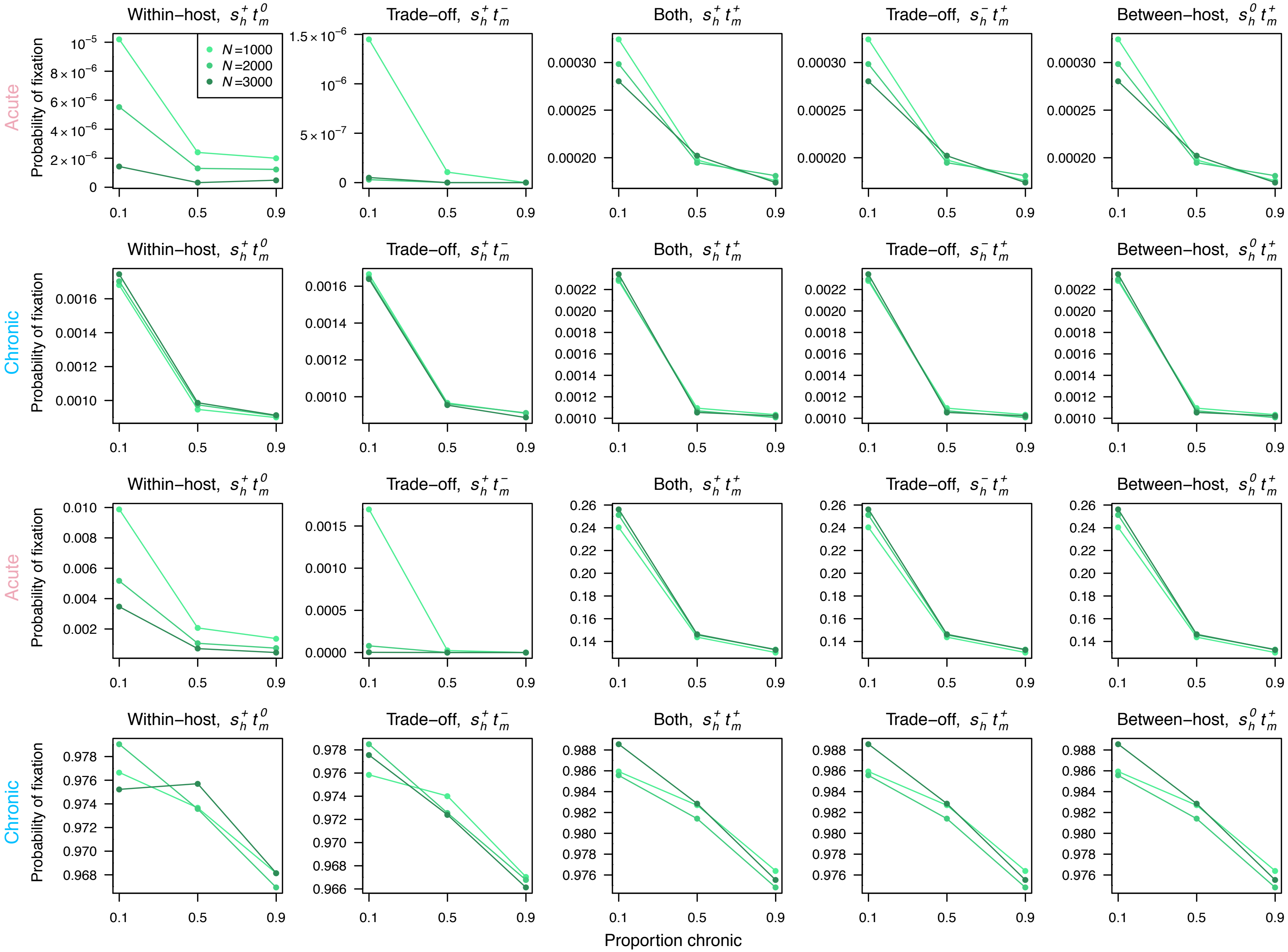

Supplementary Figure S4. The impact of prevalence on the probability of fixation. Generally, the results are qualitatively similar when the total number of infections (N), a proxy for prevalence in our model, was varied (shaded green lines) between 1000 and 3000. When the prevalence differs, the probability of fixation is qualitatively the same, except when between-host selection is advantageous but within-host selection is neutral ($s_h^0 t_m^+$ right column), which is highly influenced by stochastic frequency fluctuations within the host. More variability is also seen when the proportion of chronic infections is low (left three panels).

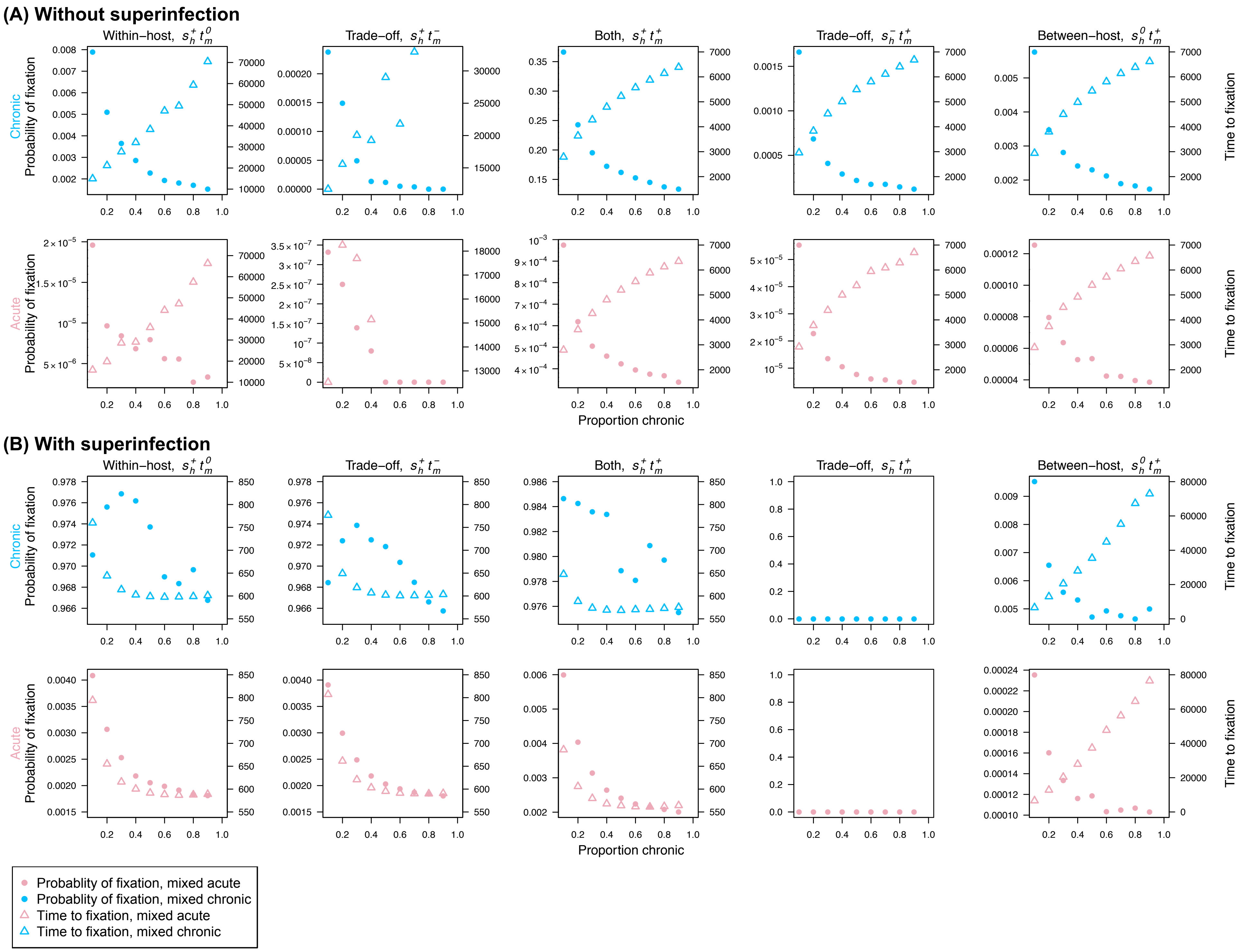

(A) Without superinfection
Within–host, $s_h^+ t_m^0$
Trade–off, $s_h^+ t_m^-$
Both, $s_h^+ t_m^+$
Trade–off, $s_h^- t_m^+$
Between–host, $s_h^0 t_m^+$
Chronic
Acute
Probability of fixation
Time to fixation
Proportion chronic
(B) With superinfection
Within–host, $s_h^+ t_m^0$
Trade–off, $s_h^+ t_m^-$
Both, $s_h^+ t_m^+$
Trade–off, $s_h^- t_m^+$
Between–host, $s_h^0 t_m^+$
Chronic
Acute
Probability of fixation
Time to fixation
Proportion chronic
Probablity of fixation, mixed acute
Probablity of fixation, mixed chronic
Time to fixation, mixed acute
Time to fixation, mixed chronic

Supplementary Figure S5. Doubling the infectiousness of acute to chronic infections does not change the negative association between the probability of fixation and the proportion of chronic infections. The probability of fixation (solid circles) and time to fixation (triangles) was determined in a mixed acute or mixed chronic model excluding (A) or including (B) superinfection.

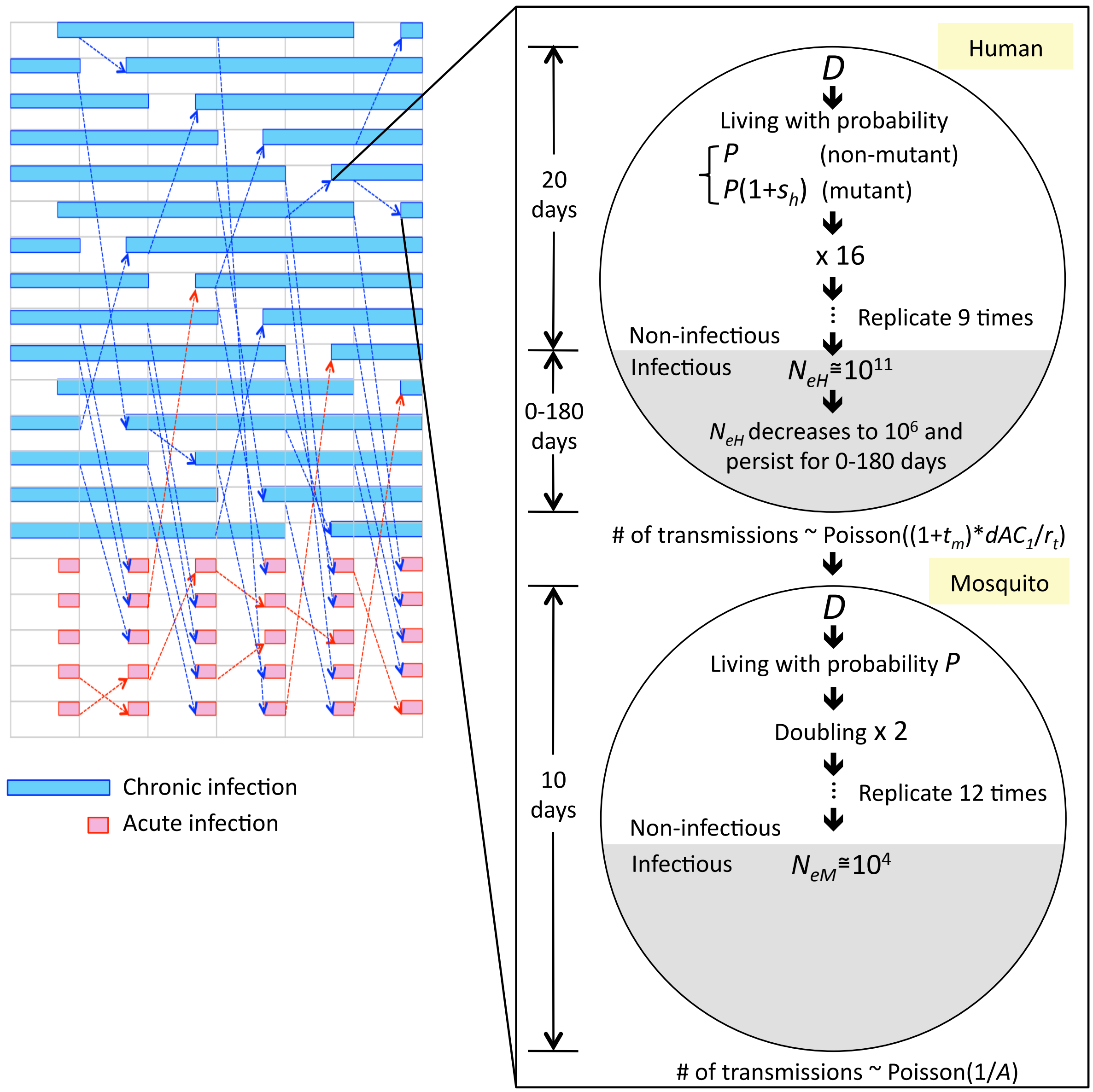

Figure S6. Schematic diagram of the model.

Supplementary Table S1. List of parameters and their baseline values

Parameters	Meaning	Baseline value	Reference
N	The number of infected human hosts	1000	
s_h	Selection coefficient within human hosts	0 (s_h^0)<br>0.1 (s_h^+)<br>-0.01 (s_h^-)	
t_m	Transmission coefficient from human to mosquito	0 (t_m^0)<br>0.1 (t_m^+)<br>-0.01 (t_m^-)	
D	The number of parasites transmitted from one host to another	10	[12,13]
A	The ratio of the number of infected mosquito hosts to the number of infected human hosts	10	[10]
P	The probability of surviving in each replication during population expansion within hosts	0.9	[13,14]
B	The relative infectiousness of acute to chronic infections	1	[1,2,11]
N_{eH}	Within-human parasite population size	~10^{11} on day 20	[12]
N_{eM}	Within-mosquito parasite population size	~10^4 on day 10	[12,13,15]
	Duration of infection in the human host	20-200 days	[16-18]
	Time to become infectious in the mosquito	10	[19,20]
	Time to become infectious in the human host	20 days	[21]

Supplementary Table S2. Within-host frequency of mutation on day 20 and day 200 without superinfection

s_h	Mutation time	Initial frequency	Average frequency on day 20	Average frequency on day 200
0.01	1st replication	6.9×10^{-3}	7.5×10^{-3}	2×10^{-2}
0.1	1st replication	6.9×10^{-3}	1.48×10^{-2}	9.88×10^{-1}
0.01	4th replication	2.33×10^{-6}	2.44×10^{-6}	7.68×10^{-5}
0.1	4th replication	2.33×10^{-6}	3.75×10^{-6}	3.6×10^{-2}

Supplementary Table S3. The comparison of the probability of fixation when controlling for the average incidence

Model	Prob. of fixation when proportion chronic = 0.5	Prob. of fixation when proportion chronic = 0.9
**Acute, without superinfection**		
Within-host, $s_h^+t_m^0$	2.40×10^{-6}	4.87×10^{-7}
Trade-off within-host, $s_h^+t_m^-$	1.06×10^{-7}	0
Both, $s_h^+t_m^+$	1.98×10^{-4}	$1.74x\ 10^{-4}$
Trade-off between-host, $s_h^-t_m^+$	3.04×10^{-6}	2.60×10^{-6}
Between-host, $s_h^0t_m^+$	2.20×10^{-5}	1.99×10^{-5}
**Acute, with superinfection**		
Within-host, $s_h^+t_m^0$	9.47×10^{-4}	9.13×10^{-4}
Trade-off within-host, $s_h^+t_m^-$	9.66×10^{-4}	8.87×10^{-4}
Both, $s_h^+t_m^+$	1.09×10^{-3}	1.02×10^{-3}
Trade-off between-host, $s_h^-t_m^+$	0	0
Between-host, $s_h^0t_m^+$	5.71×10^{-5}	5.17×10^{-5}
**Chronic, without superinfection**		
Within-host, $s_h^+t_m^0$	2.07×10^{-3}	4.50×10^{-4}
Trade-off within-host, $s_h^+t_m^-$	2.37×10^{-5}	0
Both, $s_h^+t_m^+$	1.44×10^{-1}	1.33×10^{-1}
Trade-off between-host, $s_h^-t_m^+$	1.77×10^{-4}	1.30×10^{-4}
Between-host, $s_h^0t_m^+$	1.92×10^{-3}	1.65×10^{-3}
**Chronic, with superinfection**		
Within-host, $s_h^+t_m^0$	9.74×10^{-1}	9.68×10^{-1}
Trade-off within-host, $s_h^+t_m^-$	9.74×10^{-1}	9.66×10^{-1}
Both, $s_h^+t_m^+$	9.83×10^{-1}	9.76×10^{-1}
Trade-off between-host, $s_h^-t_m^+$	0	0
Between-host, $s_h^0t_m^+$	4.98×10^{-3}	3.87×10^{-3}

References

1 Bousema, T., Okell, L., Felger, I. & Drakeley, C. Asymptomatic malaria infections: detectability, transmissibility and public health relevance. *Nat. Rev. Microbiol.* **12**, 833-840, doi:10.1038/nrmicro3364 (2014).

2 Lindblade, K. A., Steinhardt, L., Samuels, A., Kachur, S. P. & Slutsker, L. The silent threat: asymptomatic parasitemia and malaria transmission. *Expert Rev. Anti. Infect. Ther.* **11**, 623-639, doi:10.1586/eri.13.45 (2013).

3 Lin, J. T., Saunders, D. L. & Meshnick, S. R. The role of submicroscopic parasitemia in malaria transmission: what is the evidence? *Trends Parasitol.* **30**, 183-190, doi:10.1016/j.pt.2014.02.004 (2014).

4 Okell, L. C. *et al.* Factors determining the occurrence of submicroscopic malaria infections and their relevance for control. *Nat. Commun.* **3**, 1237, doi:10.1038/ncomms2241 (2012).

5 Hastings, I. M. Complex dynamics and stability of resistance to antimalarial drugs. *Parasitology* **132**, 615-624, doi:10.1017/S0031182005009790 (2006).

6 Hastings, I. M. & Watkins, W. M. Intensity of malaria transmission and the evolution of drug resistance. *Acta Trop.* **94**, 218-229, doi:10.1016/j.actatropica.2005.04.003 (2005).

7 Kim, Y., Escalante, A. A. & Schneider, K. A. A population genetic model for the initial spread of partially resistant malaria parasites under anti-malarial combination therapy and weak intrahost competition. *PloS one* **9**, e101601, doi:10.1371/journal.pone.0101601 (2014).

8 Antao, T. & Hastings, I. M. ogaraK: a population genetics simulator for malaria. *Bioinformatics* **27**, 1335-1336, doi:10.1093/bioinformatics/btr139 (2011).

9 Chang, H. H. & Hartl, D. L. Recurrent bottlenecks in the malaria life cycle obscure signals of positive selection. *Parasitology* **142 Suppl 1**, S98-S107, doi:10.1017/S0031182014000067 (2015).

10 Chang, H. H. *et al.* Malaria life cycle intensifies both natural selection and random genetic drift. *Proc. Natl. Acad. Sci. U.S.A.* **110**, 20129-20134, doi:10.1073/pnas.1319857110 (2013).

11 Churcher, T. S. *et al.* Predicting mosquito infection from Plasmodium falciparum gametocyte density and estimating the reservoir of infection. *eLife* **2**, e00626, doi:10.7554/eLife.00626 (2013).

12 Kappe, S. H., Vaughan, A. M., Boddey, J. A. & Cowman, A. F. That was then but this is now: malaria research in the time of an eradication agenda. *Science* **328**, 862-866, doi:10.1126/science.1184785 (2010).

13 White, N. J. *et al.* Malaria. *Lancet* **383**, 723-735, doi:10.1016/S0140-6736(13)60024-0 (2014).

14 Cheng, Q. *et al.* Measurement of Plasmodium falciparum growth rates in vivo: a test of malaria vaccines. *Am. J. Trop. Med. Hyg.* **57**, 495-500 (1997).

15 Rosenberg, R. & Rungsiwongse, J. The number of sporozoites produced by individual malaria oocysts. *Am. J. Trop. Med. Hyg.* **45**, 574-577 (1991).

16 Bretscher, M. T. *et al.* The distribution of Plasmodium falciparum infection durations. *Epidemics* **3**, 109-118, doi:10.1016/j.epidem.2011.03.002 (2011).

17 Eckhoff, P. P. falciparum infection durations and infectiousness are shaped by antigenic variation and innate and adaptive host immunity in a mathematical model. *PloS one* **7**, e44950, doi:10.1371/journal.pone.0044950 (2012).

18 Sama, W., Owusu-Agyei, S., Felger, I., Vounatsou, P. & Smith, T. An immigration-death model to estimate the duration of malaria infection when detectability of the parasite is imperfect. *Stat. Med.* **24**, 3269-3288, doi:10.1002/sim.2189 (2005).

19 Baton, L. A. & Ranford-Cartwright, L. C. Spreading the seeds of million-murdering death: metamorphoses of malaria in the mosquito. *Trends Parasitol.* **21**, 573-580, doi:10.1016/j.pt.2005.09.012 (2005).

20 Rosenberg, R., Wirtz, R. A., Schneider, I. & Burge, R. An estimation of the number of malaria sporozoites ejected by a feeding mosquito. *Trans. R. Soc. Trop. Med. Hyg.* **84**, 209-212 (1990).

21 Nilsson, S. K., Childs, L. M., Buckee, C. & Marti, M. Targeting Human Transmission Biology for Malaria Elimination. *PLoS Pathog.* **11**, e1004871, doi:10.1371/journal.ppat.1004871 (2015).
